# Human Macrophages Infected with a High Burden of ESAT-6-Expressing *M. tuberculosis* Undergo Caspase-1- and Cathepsin B-Independent Necrosis

**DOI:** 10.1371/journal.pone.0020302

**Published:** 2011-05-26

**Authors:** Amanda Welin, Daniel Eklund, Olle Stendahl, Maria Lerm

**Affiliations:** 1 Medical Microbiology, Department of Clinical and Experimental Medicine, Linköping University, Linköping, Sweden; 2 Center for Infectious Medicine, Department of Medicine, Karolinska Institute, Stockholm, Sweden; Hopital Raymond Poincare - Universite Versailles St. Quentin, France

## Abstract

*Mycobacterium tuberculosis* (Mtb) infects lung macrophages, which instead of killing the pathogen can be manipulated by the bacilli, creating an environment suitable for intracellular replication and spread to adjacent cells. The role of host cell death during Mtb infection is debated because the bacilli have been shown to be both anti-apoptotic, keeping the host cell alive to avoid the antimicrobial effects of apoptosis, and pro-necrotic, killing the host macrophage to allow infection of neighboring cells. Since mycobacteria activate the NLRP3 inflammasome in macrophages, we investigated whether Mtb could induce one of the recently described inflammasome-linked cell death modes pyroptosis and pyronecrosis. These are mediated through caspase-1 and cathepsin-B, respectively. Human monocyte-derived macrophages were infected with virulent (H37Rv) Mtb at a multiplicity of infection (MOI) of 1 or 10. The higher MOI resulted in strongly enhanced release of IL-1β, while a low MOI gave no IL-1β response. The infected macrophages were collected and cell viability in terms of the integrity of DNA, mitochondria and the plasma membrane was determined. We found that infection with H37Rv at MOI 10, but not MOI 1, over two days led to extensive DNA fragmentation, loss of mitochondrial membrane potential, loss of plasma membrane integrity, and HMGB1 release. Although we observed plasma membrane permeabilization and IL-1β release from infected cells, the cell death induced by Mtb was not dependent on caspase-1 or cathepsin B. It was, however, dependent on mycobacterial expression of ESAT-6. We conclude that as virulent Mtb reaches a threshold number of bacilli inside the human macrophage, ESAT-6-dependent necrosis occurs, activating caspase-1 in the process.

## Introduction

Upon inhalation, *Mycobacterium tuberculosis* (Mtb) infects alveolar macrophages in the lung and can evade host immunity to create a favorable environment for intracellular replication and subsequent spread. The significance of host cell death during Mtb infection has been debated in recent years. On the one hand, it is advantageous for the bacterium to keep the macrophage alive upon infection in order to allow sufficient time for intracellular replication and to avoid the antimicrobial effects of apoptosis. To this end, Mtb has developed mechanisms to limit macrophage apoptosis [1], [2]. On the other hand, death of the host macrophage is necessary for the bacilli to escape and infect new cells, and infection of macrophages with virulent Mtb above a certain multiplicity of infection (MOI) quickly reduces viability of the host cells, in a manner reminiscent of necrosis rather than apoptosis [3], [4], [5], [6]. Additionally, Mtb secretes early secreted antigen target 6 (ESAT-6); a protein that is encoded by the region of difference 1 (RD1) of the genome and has been shown to be a major virulence factor with membrane-lysing activity [7], [8], [9], [10].

Apoptosis, known as programmed cell death, can be induced by an intrinsic or extrinsic pathway. Both pathways lead to DNA cleavage and formation of apoptotic vesicles in a caspase-dependent manner [11], [12]. Necrosis, on the other hand, is a pro-inflammatory caspase-independent cell death mode induced by different types of stress, inflammation or microbial infection. It is characterized by disorganized DNA hydrolysis, lysosomal and mitochondrial destabilization, plasma membrane permeabilization and release of high-mobility group protein 1 (HMGB1) from the nucleus [11], [12]. Apart from these well known cell death programs, several new ones have been described in recent years [13], [14], [15], [16], [17], [18]. The different types of cell death play different roles during microbial infections in terms of host defence and microbe survival [12], [19]. Pyroptosis is a microbe-induced cell death mode where inflammasome complexes assemble and caspase-1 is activated. Activation of caspase-1 generally leads to cleavage of pre-formed proIL-1β and release of the mature cytokine. However, during pyroptosis, caspase-1 activation results in permeabilization of the plasma membrane and DNA fragmentation leading to cell death coupled with IL-1β release [13], [14], [17], [20]. Pyronecrosis is another pathogen-induced cell death mode where cell membrane permeabilization occurs independently of caspase-1, although coinciding with caspase-1 activation and IL-1β release. Pyronecrosis is dependent on cathepsin B, NACHT, LRR and PYD domains-containing protein 3 (NLRP3) and apoptosis-associated speck-like protein containing a CARD (ASC) but independent of inflammasome assembly [16], [18], [21]. It has not yet been elucidated what type of cell death is induced by a high MOI-infection with virulent Mtb in light of these recent discoveries.

We have previously shown that infection of human monocyte-derived macrophages (hMDMs) with the virulent Mtb strain H37Rv at an MOI of 10 leads to an almost complete loss of macrophage viability after three days of infection [22]. Additionally, mycobacteria have been shown to activate the NLRP3 inflammasome [23], [24], [25]. Thus, we investigated whether Mtb could induce caspase-1 or cathepsin B-dependent cell death in hMDMs, signifying pyroptosis or pyronecrosis. Despite the fact that cell death involved plasma membrane permeabilization and coincided with IL-1β release, we conclude that necrosis induced upon infection of human macrophages with virulent Mtb secreting ESAT-6 over several days is independent of caspase-1 and cathepsin B.

## Results

### Uptake and intracellular replication of H37Rv at MOI 1 and 10

**Figure 1:**
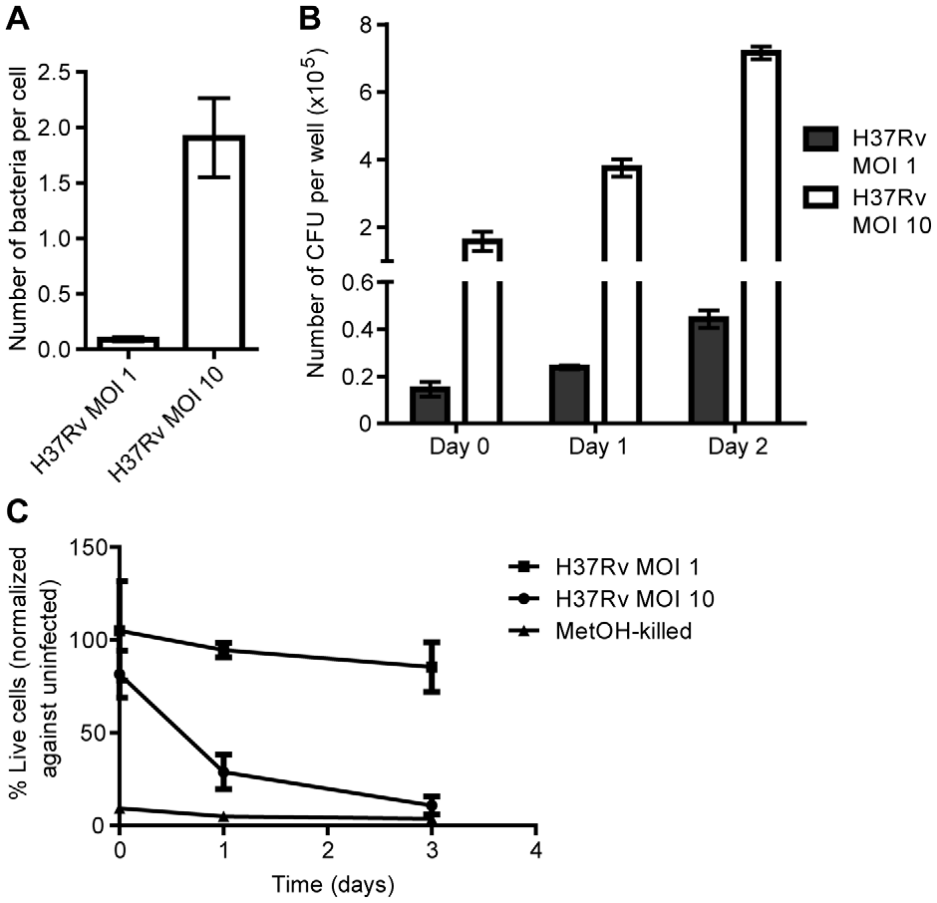
Phagocytosis, replication and cell death. hMDMs were infected with luciferase-expressing H37Rv at MOI 1 or 10 for up to three days. Bacterial numbers were assessed by luminometry, and macrophage viability by calcein AM staining and fluorescence intensity measurement. **A**) Comparison of the number of bacteria phagocytosed per cell after 4 h at the different MOIs (n = 9). **B**) Comparison of the number of intracellular H37Rv over two days at MOI 1 and 10 (n = 3). **C**) Relative viability of hMDMs infected with H37Rv at MOI 10 or 1 over three days (n = 3). The values were normalized against uninfected controls from the same reading, which were set to 100%. Values from methanol-treated cells are shown for comparison. Bar graphs show the mean and SEM.

We have previously shown that at an MOI of 10, H37Rv but not H37Ra is capable of intracellular replication in hMDMs, and that H37Rv infection leads to a loss of host cell viability within three days [22]. In order to compare bacterial uptake and replication in macrophages challenged at different MOIs, we infected hMDMs with a single-cell suspension of luciferase-expressing H37Rv at MOI 1 or 10 and measured the amount of luminescence in cell lysates over two days. Infection at MOI 1 resulted in an effective MOI of approximately one bacterium in every ten cells, while infection at MOI 10 resulted in a bacterial load of approximately two bacteria per cell (Fig. 1A). As shown in Fig. 1B, the bacilli increased in number over two days at both MOIs. The higher bacterial burden at MOI 10 correlated with loss of cell viability, as indicated by calcein AM staining of macrophages infected for up to three days (Fig. 1C). Macrophages infected at MOI 1, on the other hand, remained viable throughout the experiments (Fig. 1C).

### H37Rv-induced cell death coincides with IL-1β release

**Figure 2:**
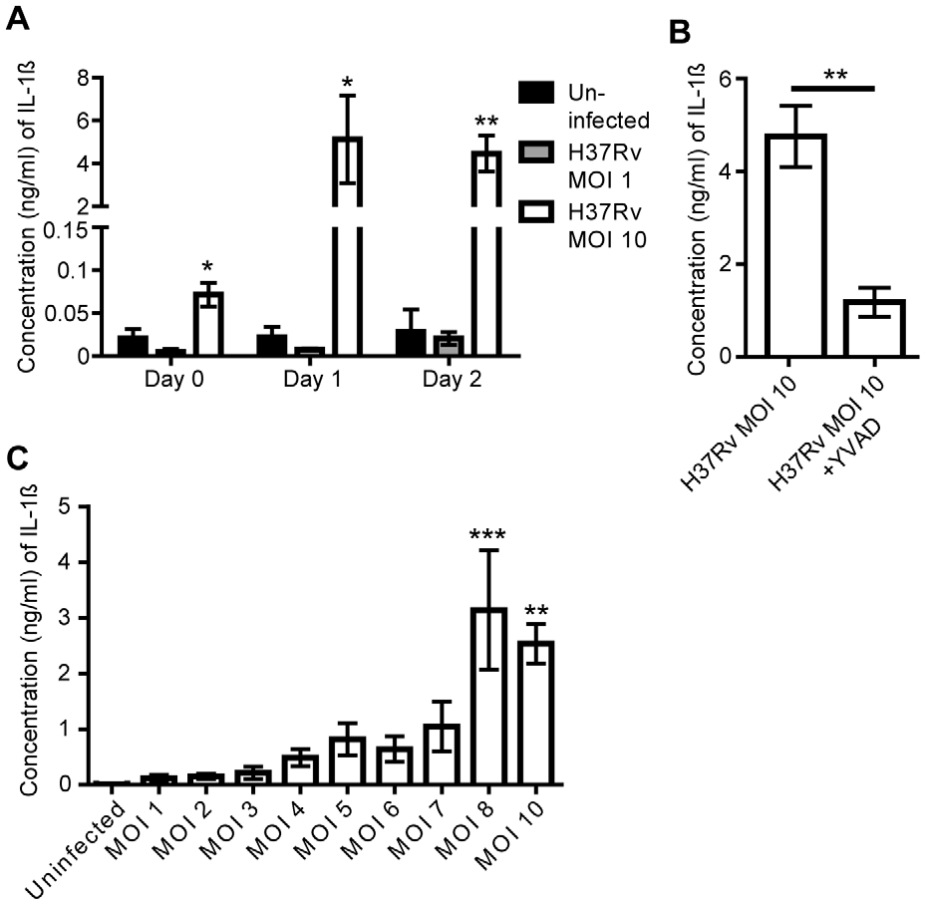
ELISA for IL-1β. hMDMs were infected with H37Rv, or left uninfected, and cell culture supernatants were collected at the indicated time points. IL-1β concentration was analyzed in triplicate by ELISA, and median values were used. **A**) IL-1β concentration in supernatants of uninfected hMDMs or hMDMs infected at MOI 1 and 10 (n = 3). **B**) IL-1β concentration in supernatants of control and YVAD-treated infected hMDMs (n = 4) after two days. **C**) IL-1β concentration in supernatants of hMDMs infected with H37Rv for two days at the indicated MOI (n = 4). Bar graphs show the mean and SEM. In A and C, the means were compared to that of uninfected cells on each day using ANOVA with Dunnett’s post-hoc test. In B, the means were compared using Student’s t-test. A statistically significant difference is denoted by *(p<0.05), **(p<0.01), or ***(p<0.001).

Pyroptosis and pyronecrosis coincide with the release of the pro-inflammatory cytokine IL-1β [12]. In order to study whether the cell death induced during intracellular replication of H37Rv was associated with release of IL-1β, we infected hMDMs for up to two days and analyzed the cell supernatants for IL-1β. H37Rv infection at MOI 10 resulted in a significant increase in the IL-1β concentration as compared to uninfected cells (p<0.05, Fig. 2A). H37Rv inoculation at MOI 1, on the other hand, did not trigger IL-1β release (Fig. 2A). IL-1β release was inhibited by addition of the caspase-1 inhibitor YVAD (p<0.01, Fig. 2B). To further investigate the effect of MOI on IL-1β release, hMDMs were infected over a range of MOIs and IL-1β was assayed. At MOI 8 and above, a significant increase in IL-1β could be detected as compared to uninfected cells (p<0.01, Fig. 2C).

### Features of cell death induced by H37Rv

**Figure 3:**
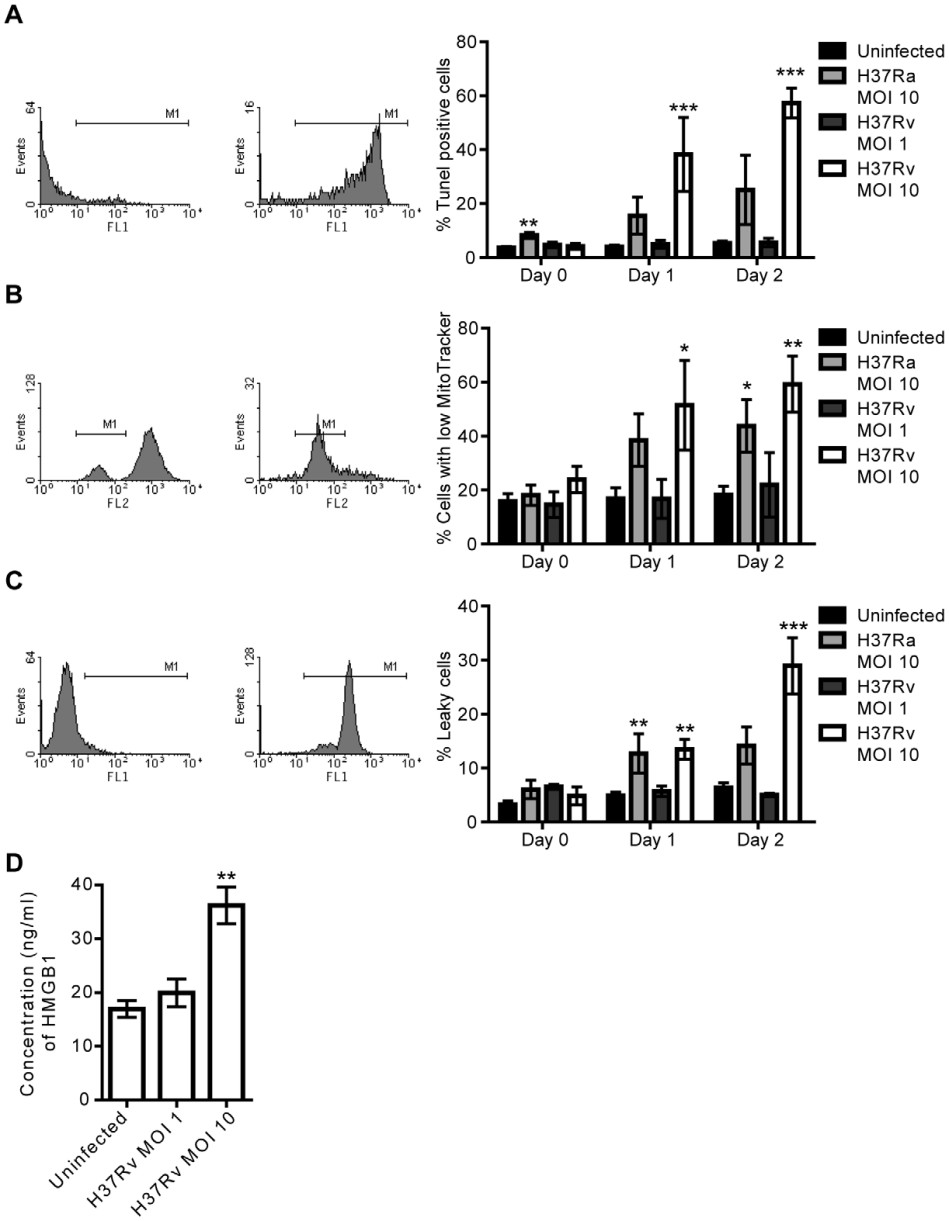
Features of cell death induced by Mtb. hMDMs were left uninfected, or infected with H37Rv at MOI 1 or 10, or H37Ra at MOI 10 over two days. Three different staining procedures were used to investigate cell death features on each day, and the cells were analyzed by flow cytometry. **A**) TUNEL analysis of DNA fragmentation. The first histogram shows representative flow cytometry data for hMDMs negative for TUNEL staining (untreated), and the second one for hMDMs positive for TUNEL staining (DNase-treated). The bar graph shows the percentage of hMDMs that were positive for TUNEL staining in uninfected or infected samples over two days (n≥5). **B**) MitoTracker analysis of mitochondrial membrane potential loss. The first histogram shows representative flow cytometry data for hMDMs with high MitoTracker staining (untreated), and the second one for hMDMs with low MitoTracker staining (UV-treated). The bar graph shows the percentage of hMDMs that had low MitoTracker staining (i.e. compromised mitochondria) in uninfected or infected samples over two days (n≥3). **C**) Plasma membrane integrity analysis. The first histogram shows representative flow cytometry data for hMDMs negative for plasma membrane leakiness staining (untreated), and the second one for hMDMs positive for plasma membrane leakiness staining (heat-treated). The bar graph shows the percentage of hMDMs that were positive for plasma membrane leakiness staining in uninfected or infected samples over two days (n≥3). **D**) HMGB1 concentration in supernatants of uninfected hMDMs or hMDMs infected with H37Rv for two days at the indicated MOI (n = 3), as measured by ELISA. Bar graphs show the mean and SEM. The means were compared to that of uninfected hMDMs on each day using ANOVA with Dunnett’s post-hoc test. A statistically significant difference from uninfected cells is denoted by *(p<0.05), **(p<0.01), or ***(p<0.001).

DNA fragmentation and increased permeability of the cell membrane are well-characterized features of pyroptosis and pyronecrosis, while contradicting results concerning changes in the mitochondrial membrane potential have been shown [16], [21], [26]. In our system, the higher bacterial burden of H37Rv inside macrophages caused cell death and release of IL-1β. In order to characterize the features of cell death induced by H37Rv, we analyzed hMDMs infected for up to two days using three different assays. Cells infected with avirulent H37Ra were analyzed for comparison since H37Ra has previously been shown to induce macrophage apoptosis, with caspase-dependent DNA fragmentation [4], [6], [27], [28]. TUNEL staining and flow cytometry analysis showed that H37Rv infection at MOI 10 caused extensive DNA fragmentation (p<0.001 at Day 1 and 2, Fig. 3A). In contrast, infection with a lower MOI of H37Rv did not induce any DNA fragmentation. H37Ra at MOI 10 induced less DNA fragmentation than H37Rv after one and two days of infection (Fig. 3A). TUNEL staining of infected cells on glass cover slips and fluorescence microscopy analysis confirmed this result (Fig. S1). Cell samples were also stained using MitoTracker to assess mitochondrial integrity. The results showed a similar pattern to the DNA fragmentation data, where H37Rv at the higher but not lower MOI led to an increase in the number of cells with compromised mitochondrial integrity (p<0.05 at Day 1 and p<0.01 at Day 2, Fig. 3B). Infection with H37Ra at MOI 10 also led to an increase in the number of cells with compromised mitochondrial potential as compared to uninfected cells (p<0.05 at Day 2, Fig. 3B). Finally, a time-dependent damage to macrophage plasma membranes could be clearly seen in H37Rv infection at MOI 10 (p<0.01 at Day 1 and p<0.001 at Day 2), while no increase in plasma membrane permeability was observed in cells with a low burden of H37Rv. Infection with H37Ra resulted in moderate cell damage (Fig. 3C). Thus, all three cell death-related features were induced by the higher bacterial burden of H37Rv. Furthermore, H37Rv infection at MOI 10 led to elevated levels of HMGB1 in the cell culture supernatant (p<0.01, Fig. 3D).

### H37Rv-induced cell death is caspase- and cathepsin B-independent

**Figure 4:**
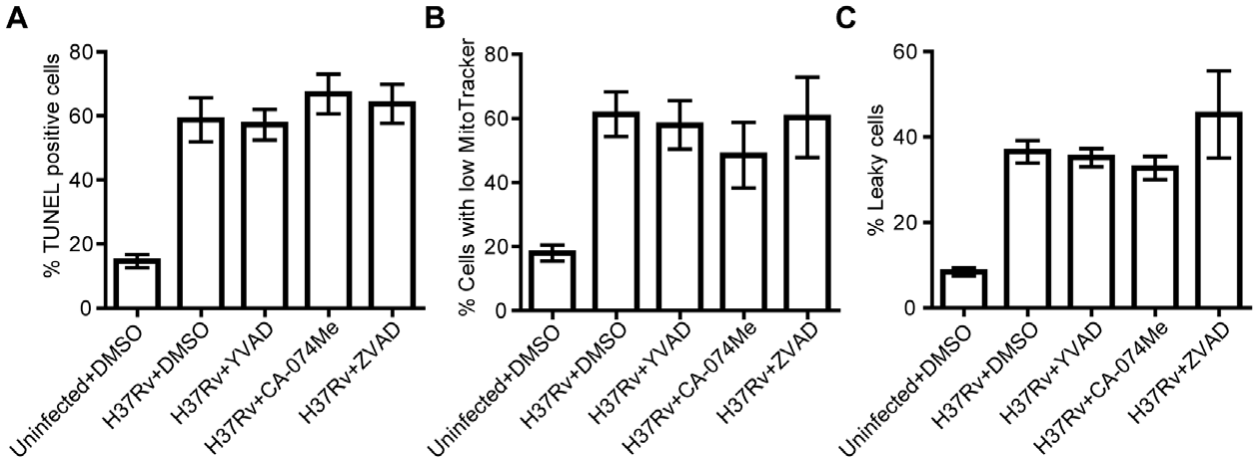
Effect of inhibitors on cell death. hMDMs were treated with DMSO (1:1000) or inhibitors (50 µM) dissolved in DMSO, and infected with H37Rv at MOI 10 or left uninfected. After two days, the features of cell death were analyzed using three different staining procedures and flow cytometry. **A**) TUNEL analysis of DNA fragmentation (n≥4). **B**) MitoTracker analysis of mitochondrial membrane potential loss (n≥4). **C**) Plasma membrane integrity analysis (n≥4). Bar graphs show the mean and SEM. The means were compared to that of H37Rv-infected and DMSO-treated hMDMs using ANOVA with Dunnett’s post-hoc test.

H37Rv was shown to cause extensive macrophage cell death at MOI 10 using the flow cytometry-based assays above. The facts that the cell death involved loss of plasma membrane integrity, DNA fragmentation, and coincided with IL-1β release are consistent with features of inflammasome-related cell death. However, the experiments did not reveal the mechanism by which cell death was induced. To investigate if cell death caused by H37Rv could be blocked, different inhibitors of cellular enzymes were added to cells during infection. The analysis showed that neither the caspase-1 inhibitor YVAD, the cathepsin B inhibitor CA-074Me, nor the pan-caspase inhibitor ZVAD had any significant effect on DNA fragmentation (Fig. 4A), loss of mitochondrial potential (Fig. 4B) or compromised plasma membrane integrity (Fig. 4C) after two days of infection. Furthermore, treatment of the hMDMs with YVAD or CA-074Me did not affect infection-induced HMGB1 release (Fig. S2). Control experiments showed that incubation of uninfected hMDMs for 24 h with the inhibitors did not influence cell viability (no more than 2% increase in the proportion of TUNEL-positive, MitoTracker-low or leaky cells as compared to control, Fig. S3A–C). On the other hand, the concentration of ZVAD used effectively reduced DNA fragmentation upon exposure of hMDMs to UV light, while YVAD did not affect UV-induced DNA fragmentation (Fig. S4A–C). The concentration of CA-074Me used effectively reduced staurosporine-induced apoptosis (Fig. S4D), as previously reported [29]. Thus, we show that the cell death mode induced by H37Rv at MOI 10 was independent of caspase-1, other caspases, and cathepsin B, indicating that Mtb did not induce pyroptosis, pyronecrosis, or apoptosis.

### Cell death induced by H37Rv at MOI 10 is dependent on ESAT-6

**Figure 5:**
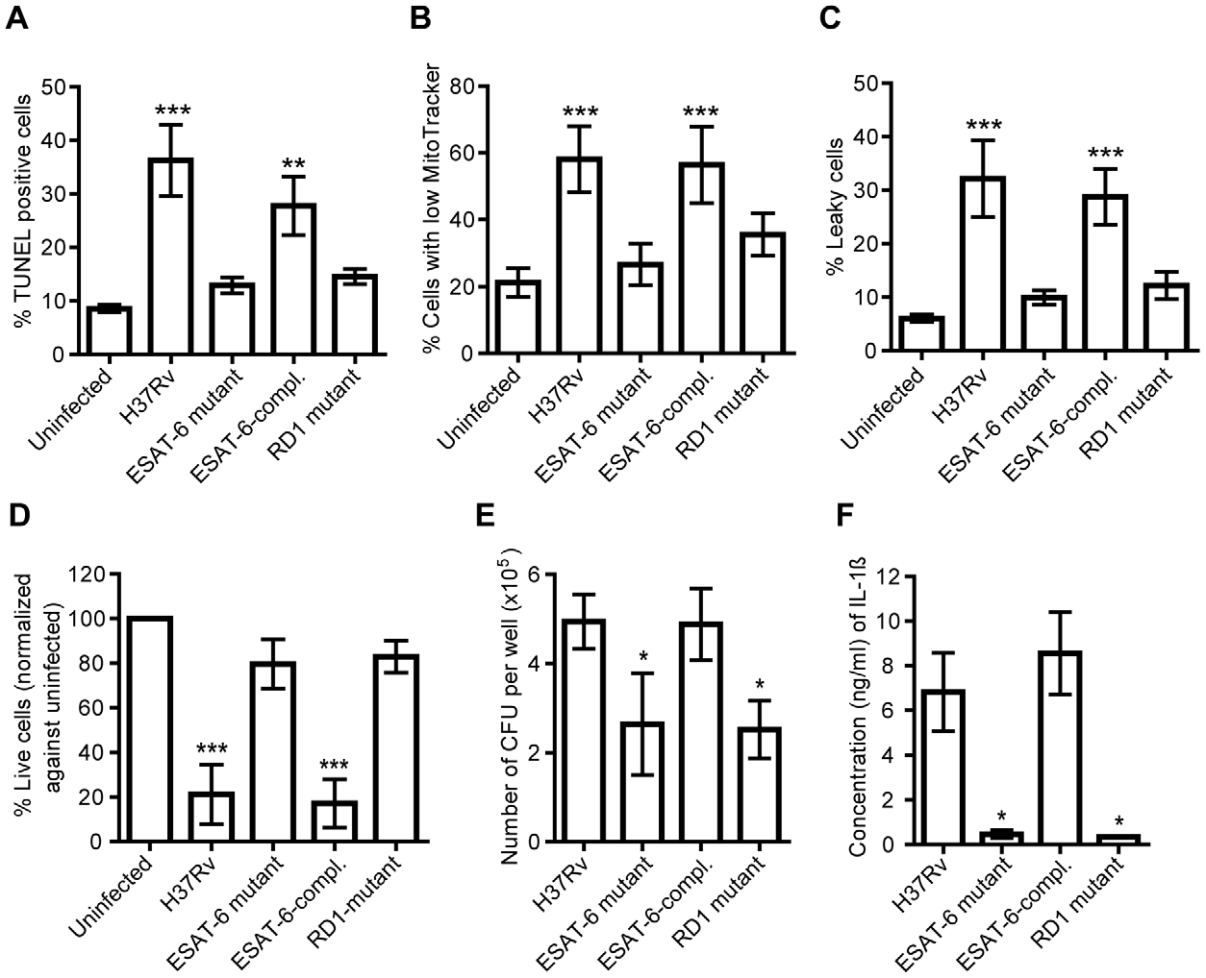
The role of ESAT-6 in macrophage necrosis. hMDMs were left uninfected or infected at the indicated MOI with H37Rv, an ESAT-6 mutant, a complemented ESAT-6 mutant (ESAT-6-compl.), or an RD1 mutant, and cell death was assessed by the three different staining kits after two days, and analyzed by flow cytometry, or by calcein staining. Alternatively, infected cells were lysed, dilutions were plated for viable count, and the number of CFU was enumerated. Cell culture supernatants were collected and IL-1β was assayed by ELISA. **A**) TUNEL analysis of DNA fragmentation after infection at MOI 10 (n = 6). **B**) MitoTracker analysis of mitochondrial membrane potential loss after infection at MOI 10 (n = 6). **C**) Plasma membrane integrity analysis after infection at MOI 10 (n = 6). **D**) Relative viability of hMDMs infected for two days with the different strains at MOI 40, as analyzed by calcein staining (n = 4). The values were normalized against uninfected controls from the same reading, which were set to 100%. **E**) The number of CFU per well after infection with the different strains at MOI 10 for two days, as determined by viable count analysis (n = 5). **F**) IL-1β concentration in cell culture supernatants from hMDMs infected for two days with the different strains at MOI 10 (n = 3). Bar graphs show the mean and SEM. The means were compared to that of uninfected hMDMs (A-D) or H37Rv-infected hMDMs (E-F) using ANOVA with Dunnett’s post-hoc test. A statistically significant difference is denoted by *(p<0.05), **(p<0.01), or ***(p<0.001).

We established that H37Rv-induced cell death in hMDMs was not executed by any of the cell death signals tested above. However, we hypothesized that induction of cell death might be dependent on bacterial factors as well as bacterial load. To elucidate this we used ESAT-6-deficient mutants of H37Rv. hMDMs were infected at MOI 10 with wild type H37Rv, an ESAT-6 deletion mutant (H37Rv:Δ3875), an ESAT-6 complemented strain (H37Rv:Δ3875:pMH406), and an RD1 deletion mutant (H37Rv:ΔRD1) [9] and analyzed by all three cell death assays. Infection with the parental H37Rv strain induced significant DNA fragmentation (p<0.001), mitochondrial membrane permeabilization (p<0.001) and plasma membrane integrity loss (p<0.001), as compared to uninfected controls, while the ESAT-6 or RD1 mutant did not induce any cell death features. Complementation of ESAT-6 significantly restored the capability to induce DNA fragmentation (p<0.01), mitochondrial membrane permeabilization (p<0.001) and plasma membrane integrity loss (p<0.001), as compared to the uninfected controls (Fig. 5A–C). Furthermore, the increased concentration of HMGB1 in the cell culture supernatant observed after H37Rv infection was dependent on an intact ESAT-6 gene (Fig. S2). To investigate whether cell death could be triggered by the mutant strains when the bacterial load was increased further, hMDMs were infected at an MOI of 40, and cell viability was assessed by the calcein assay. Even at MOI 40, however, the ESAT-6 and RD1 mutants failed to induce cell death (Fig. 5D). To investigate growth of the different strains upon challenge of hMDMs, cells infected for two days were lysed and the number of colony forming units (CFU) was enumerated after two to three weeks. This showed that both the ESAT-6 (p<0.05) and RD1 (p<0.05) mutants were significantly deficient in total growth as compared to the parental H37Rv strain. When the ESAT-6-complemented strain was used to infect hMDMs, however, the replication ability was restored (Fig. 5E). Thus, the cell death induced by H37Rv was directly or indirectly dependent on a functional RD1 region. Furthermore, as shown in Fig. 5F, infection of hMDMs with the ESAT-6 (p<0.05) or RD1 (p<0.05) mutant strain resulted in diminished secretion of IL-1β from the cells, as compared to the wild type strain.

## Discussion

Necrosis in the center of tuberculous granulomas is a hallmark of human tuberculosis, while apoptosis is a general host strategy for killing and removal of intracellular pathogens. The presence of a necrotic core in the granuloma suggests an important role for host cell death in the pathogenesis of tuberculosis, but the specific characteristics of cell death induced by Mtb have not been fully elucidated. We set out to study whether Mtb infection of human macrophages can lead to one of the recently described inflammasome-associated cell death types.

We found that infection of hMDMs with virulent H37Rv at MOI 10 resulted in extensive host cell death within three days, while cells remained unaffected by infection at MOI 1. Although the number of intracellular bacteria increased over time at both MOI 1 and 10, the actual number of bacilli in the samples differed greatly between the two MOIs. Our data suggest that the absolute bacterial burden is a determinant of the outcome for the cell in terms of cell viability. Previous studies have also shown that a high intracellular burden of Mtb leads to cytolysis [4], [6], [30], [31], [32], an event which may enable spread to neighboring cells [8], [9]. The ability of virulent Mtb to inhibit apoptosis has been documented several times [1], [2], [33], while avirulent Mtb has been shown to induce TNF-α-mediated apoptosis, which is accompanied by killing of the pathogen [27], [34], [35], [36], [37]. The diversity of the obtained results may be due to differences in experimental setup, including cell type and origin, Mtb strain, MOI, and the time points studied, as reviewed by Lee et al. [38]. This gives rise to the idea that virulent Mtb is anti-apoptotic at low intracellular burden while above a certain threshold in bacterial numbers, the bacilli induce cytolysis to promote spread to adjacent cells. A previous study using mouse macrophages have suggested a threshold of 20 bacilli per cell [4], while our results point to a lower threshold in hMDMs.

H37Rv infection has previously been shown to induce a strong IL-1β response in macrophages [23], [24], [25], [39]. We show here that the caspase-1-dependent IL-1β secretion from Mtb-infected hMDMs was only triggered by the higher MOI of H37Rv, giving a large increase of IL-1β in the cell culture supernatant. Interestingly, a clear tipping point was observed at MOI 8, suggesting that excessive inflammasome activation occurs beyond a critical bacterial load. The induction of IL-1β was dependent on the secretion of ESAT-6, which is also in line with other recent studies [23], [24], [25]. ESAT-6 and the secretion system ESX-1 through which ESAT-6 is secreted are both encoded by the RD1 region of the Mtb genome [8]. A recent study using RD1 mutants suggested that factors expressed in the RD1 locus cause pore formation in the cell membrane, followed by potassium efflux leading to inflammasome assembly, caspase-1 activation and IL-1β release [40]. Our study supports the notion that ESAT-6 is a trigger for inflammasome assembly and IL-1β release, possibly through its pore-forming activity.

Since Mtb-induced cell death coincided with the release of IL-1β, we further investigated the cell death with respect to characteristics of pyroptosis and pyronecrosis. Infection of hMDMs with H37Rv at MOI 10 led to DNA fragmentation and loss of mitochondrial membrane potential. These features are indicative of apoptosis, a type of cell death which leads to caspase-dependent chromatin condensation, DNA fragmentation, mitochondrial permeabilization, and formation of confined apoptotic bodies [11], [12], [26]. However, infection with H37Rv also gave rise to increased permeability of the plasma membrane as well as increased release of the inflammatory mediator HMGB1, indicating that the main pathway of cell death was not apoptosis. All the known necrosis-like cell death pathways are coupled with HMGB1 release [12], [15], [16], [18], [41], [42], and it has been shown previously that macrophage infection with Mtb leads to release of HMGB1, fuelling the inflammatory response [43], [44].

Infection of hMDMs for two days with the avirulent H37Ra strain at MOI 10 also resulted in DNA fragmentation and loss of mitochondrial membrane potential, but modest plasma membrane permeabilization. These features are consistent with the induction of apoptosis in H37Ra-infected macrophages [4], [6], [27]. The difference observed between the strains may be due to the ability of H37Rv to replicate intracellularly, while a mutation in the *phoP* gene has rendered H37Ra unable to adapt to the intracellular environment and thus unable to replicate efficiently [45], [46], [47].

To ensure that the cytolysis observed with H37Rv was not post-apoptotic necrosis, we infected macrophages in the presence of a pan-caspase inhibitor, and found that the cell death was caspase-independent. The loss of plasma membrane integrity observed during H37Rv-infection together with DNA fragmentation and IL-1β release is consistent with pyroptosis or pyronecrosis [13], [14], [15], [16], [17], [20], [21]. However, pyroptosis is dependent on caspase-1 and pyronecrosis on cathepsin B. Inhibition of these enzymes in our system did not affect the cell death induced by H37Rv. In a previous study, inhibition of cathepsin B had a slight effect on the cell membrane integrity loss induced by Mtb at MOI 25 [4], but this was not observed in the present study. Our data thus suggest that the observed Mtb-induced cell death was not pyroptosis or pyronecrosis. Since we were also unable to inhibit the cell death induced by H37Rv by blocking the action of pro-apoptotic caspases, we conclude that H37Rv induces necrosis in hMDMs. This is consistent with the morphological features observed [11], [12], [26], including DNA fragmentation, which is a hallmark of apoptosis, but has also been observed in a disorganized fashion during necrosis [12], [48], [49]. Our conclusion that macrophage necrosis is induced by a high intracellular burden of H37Rv is consistent with several previous studies [4], [6], [30], [31], [32]. We speculate that although it may be possible to influence specific features of Mtb-induced cell death using inhibitors, as others have done [4], [6], [50], [51], cell death is an inevitable outcome in our infection model at MOI 10.

It has previously been shown that the secreted mycobacterial protein ESAT-6 in its purified form induces cell death in macrophages [5], and that the protein has membrane-lysing activity [7], [8], [9], [10]. This makes ESAT-6 an attractive candidate as the bacterial factor responsible for necrosis. We found that deletion of the gene encoding ESAT-6 or of the RD1 region led to abrogation of the necrosis-inducing effect of Mtb as well as diminished IL-1β secretion by the host macrophage, suggesting that ESAT-6 was causing necrosis. This was true even at a very high MOI, suggesting that the mere presence of a large number of bacilli was not sufficient to induce cell death. Evaluation of the number of CFU after two days of hMDM-infection with the wild type and mutant strains showed that both the ESAT-6 and RD1 mutant displayed reduced bacterial numbers as compared to wild type H37Rv, questioning the direct effect of ESAT-6. However, a previous study showed that these mutants were capable of intracellular replication, but not of cell-to-cell spread, ultimately resulting in lower total bacterial counts [9]. We hypothesize that ESAT-6 secretion is necessary for the necrosis, which in turn is a prerequisite for subsequent cell-to-cell spread [8], [9]. This is consistent with data obtained with *M. marinum*, where RD1-deficient mutants failed to induce cell death and thereby failed to infect newly arriving macrophages [52]. Another study in a mouse model showed that *M. marinum* induces RD1-dependent tissue damage through necrosis as well as excessive production of IL-1β, but that the induction of necrosis did not involve the inflammasome components ASC or NLRP3 [23]. Rather, cell death and IL-1β release were parallel events, which is consistent with our results with Mtb. IL-1β is crucial for control of Mtb in mice [53], [54], while the protective role of the inflammasome has not been confirmed [55], [56] and inflammasome activation can cause excessive inflammation and tissue damage [23]. This highlights the importance of a tightly regulated response.

Altogether, our results show that hMDMs infected with virulent Mtb undergo necrosis in an ESAT-6-dependent manner. Cell death is not achieved through caspase-1 or cathepsin B activation although it is accompanied by IL-1β secretion. Our current hypothesis is that when Mtb is present intracellularly at low numbers, the host cell is kept alive to maintain the bacterial niche. Above a certain threshold, the bacilli benefit from escaping the initially infected host cell as this leads to dissemination. Thus, they induce macrophage necrosis via ESAT-6, overriding the apoptotic response favored by the host. The inflammasome is activated in the process, giving rise to high levels of IL-1β, which increases the inflammatory response to infection. The process contributes to the host response but also to the formation of the necrotizing granuloma centre, which is a prerequisite for spread of Mtb. Future studies focusing on the cellular mechanisms and signaling pathways behind ESAT-6-induced necrosis would be of great interest.

## Materials and Methods

### Bacteria and macrophage culture

H37Rv and H37Ra were obtained from ATCC and mutant strains H37Rv:Δ3875, H37Rv:ΔRD1 and H37Rv:Δ3875:pMH406 were a kind gift from Prof. David Sherman [9]. For luciferase expression, H37Rv was transformed with the pSMT1 plasmid [22], [57]. All strains were grown in 7H9 broth supplemented with 0.05% Tween-80, 0.5% glycerol and ADC enrichment (BD) for two to three weeks before passageing and culturing for an additional 7 days prior to experiments. All the strains used in the study behaved similarly in terms of growth *in vitro*, and the number of CFU correlated well with luciferase expression after infection of hMDMs (Fig. S5A–D). hMDMs were cultured by separating monocytes from peripheral blood obtained from healthy blood donors, as previously described [22], [58]. In brief, monocytes were obtained by layering blood onto a density gradient, followed by centrifugation and isolation of the monocyte/lymphocyte fraction. Cells were seeded in culture flasks and allowed to adhere for 2 h before non-adherent lymphocytes were washed away. The monocytes were allowed to differentiate into hMDMs for 5–8 days in Dulbecco’s modified Eagle’s medium (DMEM) containing 25 mM Hepes, 100 U/ml penicillin, 100 µg/ml streptomycin and 10% active human serum. One day prior to infection, cells were trypsinized and reseeded in 6-well plates (1×10^6^ cells/well) or 96-well plates (1×10^5^ cells per well) in antibiotic-free DMEM with serum.

### Experimental infection

Bacteria were prepared, as previously described [22], by washing the bacterial suspension in PBS with 0.05% Tween-80 and passage through a 27 gauge needle. This was repeated and the bacteria were resuspended in DMEM without additives (to obtain non-opsonic conditions). The concentration was determined by measuring optical density (OD_600_) as a function of CFU. The single-cell suspension of bacteria was added to control cells or cells pre-incubated for 2 h with pan-caspase inhibitor Z-VAD-FMK (Sigma-Aldrich, 50 µM) [5], caspase-1 inhibitor Ac-YVAD-CMK (Cayman Chemical, 50 µM) [59] or cathepsin B inhibitor CA-074Me (Sigma-Aldrich, 50 µM) [4], at the indicated MOI. The cells were allowed to phagocytose the bacteria for 1 h at 37°C before non-phagocytosed bacteria were removed and the cells were incubated in antibiotic-free DMEM with serum for 4 h (Day 0) or the indicated number of days. Inhibitors were added again after infection and were present throughout the experiment. Both adherent and detached cells were collected at the indicated time points for the cell death assays, and cell supernatant aliquots were frozen at -70°C for cytokine analysis. Alternatively, the cells were subjected to a luciferase assay or viable count to assess intracellular bacterial growth, or a calcein assay to evaluate general cell viability.

### Luciferase assay

In order to quantify intracellular bacterial replication of H37Rv, a luciferase-based method was used, as previously described [22]. Briefly, hMDMs seeded in white 96-well culture plates were infected in triplicates at MOI 10 or 1 with H37Rv constitutively expressing *Vibrio harvey* luciferase for the indicated times, before hypotonic lysis of the cells. Luminescence, which was linearly proportional to the number of viable bacilli in the sample, was measured after addition of substrate (1% decanal, Sigma-Aldrich) using a Modulus Microplate Reader (Turner Biosystems). The median of each triplicate was used, and the number of bacilli was obtained using the formula y = 0.5647x, where one luminescence unit corresponded to approximately two bacteria [22].

### Calcein assay

In order to assess the relative number of viable macrophages throughout the infection, hMDMs seeded in triplicate in black 96-well plates were infected as described above, incubated for the indicated times, washed three times with PBS, and incubated with 4 µM calcein acetoxymethyl (calcein AM, Molecular Probes) at RT for 30 min, as previously described [22]. Viable cells convert non-fluorescent, cell-permeable calcein AM to highly fluorescent calcein. Fluorescence, thus corresponding to the number of viable cells, was measured in the Modulus Microplate Reader using a 490 nm excitation filter. Methanol-treated cells were used as negative, and non-infected cells as positive controls. The median of each triplicate was used and the values were normalized against the value for uninfected cells of each time point.

### IL-1β ELISA

ELISA MAX™ Deluxe (Biolegend) was used to measure the concentration of IL-1β in culture supernatants, according to manufacturer's instructions. Briefly, plates pre-coated overnight with IL-1β-specific antibodies were blocked for 1 h. Standards and diluted samples were added in triplicates and left to incubate for 2 h, followed by addition of a secondary detection antibody and incubation for 1 h. To allow detection, avidin-horseradish peroxidase (HRP) was added for 30 min. Lastly, HRP substrate tetramethylbenzidine was added for 30 min before the reaction was stopped by 2N H_2_SO_4_ and absorbance was measured at 450 and 570 nm with the Modulus Microplate Reader. Based on the optical density values of the standard, the concentration of IL-1β could be calculated, and the median of each triplicate was used.

### TUNEL assay

The hMDMs were collected and washed once in PBS (5 min, 600× *g*) before being fixed in 4% paraformaldehyde (PFA) for 30 min at RT. After an additional wash step, cells were permeabilized by adding permeabilization solution (0.1% Triton X-100, 0.1% sodium citrate in H_2_0) for 2 min on ice and washed (5 min, 800× *g*). DNA strand breaks were labelled by adding terminal deoxynucleotidyl transferase-mediated dUTP nick end labelling (TUNEL) reaction mixture, according to the manufacturer’s instructions (*In Situ* Cell Death Detection Kit, Roche Applied Science). The reaction mixture contained terminal deoxynucleotidyl transferase, which adds fluorescein-labeled nucleotide polymers to the free 3’-OH DNA ends of fragmented DNA. Samples were incubated for 60 min at 37°C, washed and analyzed by flow cytometry (BD FACS Calibur, Becton Dickinson). Cells displaying fluorescence intensity above background levels were classified as positive. As a positive control, hMDMs were treated for 10 min with 40 U/ml of DNase I (Applied Biosystems) at RT prior to labeling (typically giving >60% positive cells).

### Mitochondrial integrity assay

To analyze loss of mitochondrial integrity, cells were labeled with fixable MitoTracker Red CMXRos (Invitrogen), previously used to study the loss of mitochondrial activity and apoptosis [60], [61]. Staining was performed as instructed by the manufacturer, by washing the hMDMs in PBS (5 min, 600× *g*) before incubation with 500 nM pre-warmed MitoTracker Red CMXRos for 30 min at 37°C. After incubation, cells were washed and fixed in 4% PFA for 30 min at RT. Fixed cells were subjected to a final wash (5 min, 800× *g*) before suspension in PBS and analysis by flow cytometry. Loss of mitochondrial integrity could be observed as low or absent MitoTracker Red CMXRos staining (fluorescence intensity below that of healthy cells). As a positive control, hMDMs treated with UV light at a distance of 5 cm for 7.5 min followed by incubation over night were used (typically giving >50% cells with low/absent fluorescence intensity).

### Plasma membrane integrity assay

To evaluate whether or not the integrity of the plasma membrane of the hMDMs was compromised, cells were stained with the Green LIVE/DEAD Fixable Dead Cell Stain (Invitrogen). The reactive dye reacts with cellular amines, leading to low staining of live cells (only cell-surface amines) and bright staining of permeable cells (intracellular and cell-surface amines). Cell samples were washed in PBS (5 min, 600× *g*) and stained for 30 min at RT, according to the manufacturer's instructions. After a subsequent wash, cells were fixed in 4% PFA (30 min at RT) before being washed again (5 min, 800× *g*) and resuspended in PBS. Analysis was performed by flow cytometry, where cells with brighter fluorescence intensity than that of healthy cells were considered positive. As a positive control, hMDMs were incubated at 60°C for 10 min prior to staining (typically giving >90% positive cells).

### HMGB1 ELISA

HMGB1 ELISA (IBL) was used to measure the concentration of HMGB1 in cell culture supernatants, according to manufacturer's instructions. Standards and diluted samples were added in triplicates to plates pre-coated with HMGB1-specific antibodies, and incubated overnight, before addition of a HRP-labeled secondary detection antibody and incubation for 2 h. Tetramethylbenzidine solution was added for 30 min before the reaction was stopped by 0.35 M H_2_SO_4_ and absorbance was measured at 450 and 570 nm with the Modulus Microplate Reader. Based on the optical density values of the standard, the concentration of HMGB1 could be calculated, and the median of each triplicate was used.

### CFU assay

To measure intracellular growth of bacteria not expressing luciferase, hMDMs were seeded in triplicates in 96-well culture plates (1×10^5^ cells per well) and infected as described above. At the indicated time points, cell lysates (hypotonic lysis in sterile water) were subjected to serial dilutions and plated on Middlebrook 7H10 agar plates. Plates were sealed with parafilm and incubated for two to three weeks before enumeration of the number of colonies. The median of each triplicate was used.

### Statistical analysis

For statistical analysis of two groups of data, unpaired Student’s t-test was used. For statistical analysis of more than two groups, an ANOVA with Dunnett’s post-hoc test was performed, comparing the means to that of the control group. When data was matched, a repeated measures ANOVA was used. Analyses were performed using GraphPad Prism 5, and p values of ≤0.05 were considered significant.

## Supporting Information

Figure S1 **Fluorescence microscopy images of TUNEL-stained hMDMs.** hMDMs on glass cover slips were infected with H37Ra or H37Rv at MOI 10, or left uninfected, fixed after two days of infection, and fragmented DNA was visualized by TUNEL staining. As a positive control, the hMDMs were treated with DNase prior to staining. Images were acquired using a fluorescence microscope, with the same settings for all samples. The images are representative of two independent experiments.
(TIF)

Figure S2 **Increased HMGB1 release upon H37Rv infection is independent of caspase-1 and cathepsin B, but dependent on an intact ESAT-6 gene.** hMDMs treated with DMSO (1:1000), YVAD, or CA-074Me were infected with H37Rv for two days at MOI 10. Alternatively, hMDMs were left uninfected or were infected at MOI 10 for two days with H37Rv or an ESAT-6 deletion mutant. The cell culture supernatants were then assayed for HMGB1 using ELISA (n = 3). The bar graph shows the mean HMGB1 concentration and the SEM.
(TIF)

Figure S3 **YVAD, CA-074Me and ZVAD do not affect macrophage viability.** hMDMs were left untreated or were treated for 24 h with DMSO (1:1000) or the inhibitors at the concentration used in the manuscript. Three different staining procedures were used to investigate cell death features, and the cells were analyzed by flow cytometry. **A**) TUNEL analysis of DNA fragmentation. The bar graph shows the percentage of hMDMs that were positive for TUNEL staining (n = 1). **B**) MitoTracker analysis of mitochondrial membrane potential loss. The bar graph shows the percentage of hMDMs that had low MitoTracker staining (i.e. compromised mitochondria) (n = 1). **C**) Plasma membrane integrity analysis. The bar graph shows the percentage of hMDMs that were positive for plasma membrane leakiness staining (n = 1).
(TIF)

Figure S4 **ZVAD and CA-074Me, but not YVAD, reduce UV- or staurosporine-induced apoptosis.** hMDMs were treated with DMSO (1:1000) or inhibitors at the indicated concentration, and apoptosis was induced by UV light or by treatment with staurosporine for 18 h at the indicated concentration. Apoptosis was then assessed by TUNEL or Annexin V staining. **A**) Microscopy-based TUNEL analysis of DNA fragmentation after ZVAD and UV treatment. The bar graph shows the percentage of hMDMs that were positive for TUNEL staining (n = 1). **B**) Flow cytometry-based TUNEL analysis of DNA fragmentation after ZVAD and UV treatment. The bar graph shows the percentage of hMDMs that were positive for TUNEL staining (n = 1). **C**) Flow cytometry-based TUNEL analysis of DNA fragmentation after YVAD and UV treatment. The bar graph shows the percentage of hMDMs that were positive for TUNEL staining (n = 1). **D**) Flow cytometry-based Annexin V analysis of phosphatidyl serine externalization after CA-074Me and staurosporine (STS) treatment. Staining was performed using TACS Annexin V-FITC Apoptosis Detection Kit (R&D Systems) according to the manufacturer’s instruction. The bar graph shows the percentage of hMDMs that were positive for Annexin V staining (n = 1).
(TIF)

Figure S5 **Similarity in growth between Mtb strains, and correlation between viable count and luciferase activity upon phagocytosis.** **A**) 7H9 broth was inoculated with the different strains and the increase in bacterial numbers was assessed by OD_600_ at the indicated time points. The OD value was normalized against the Day 0 value for each time point and strain (n = 1). **B**) 7H9 broth was inoculated with the different strains, serial dilutions were plated at the indicated time points, and the number of CFU was enumerated after 2-3 weeks. The mean and SEM of triplicate plates is shown (n = 1). **C**) hMDMs were infected with H37Rv or luciferase-expressing H37Rv (H37Rv lux) at MOI 10, the macrophages were lysed, diluted and plated at the indicated time points, and the number of colonies on the plate was determined after 2-3 weeks. For H37Rv lux, the mean and SEM is shown (n = 6), and for H37Rv, one experiment is shown. **D**) hMDMs were infected with H37Rv lux at MOI 1, the macrophages were lysed after 4 h, and the number of CFU in the sample was determined by measuring luminescence (Lux) and converting the value to CFU, or by plating and determining the number of CFU by viable count (VC). The mean and SEM of the triplicates is shown (n = 1).
(TIF)
