# Supplementary figures and images for: Human Macrophages Infected with a High Burden of ESAT-6-Expressing M. tuberculosis Undergo Caspase-1- and Cathepsin B-Independent Necrosis
Source: PLoS One. 2011 May 26;6(5):e20302. doi: 10.1371/journal.pone.0020302 (PMC3102687; doi:10.1371/journal.pone.0020302)

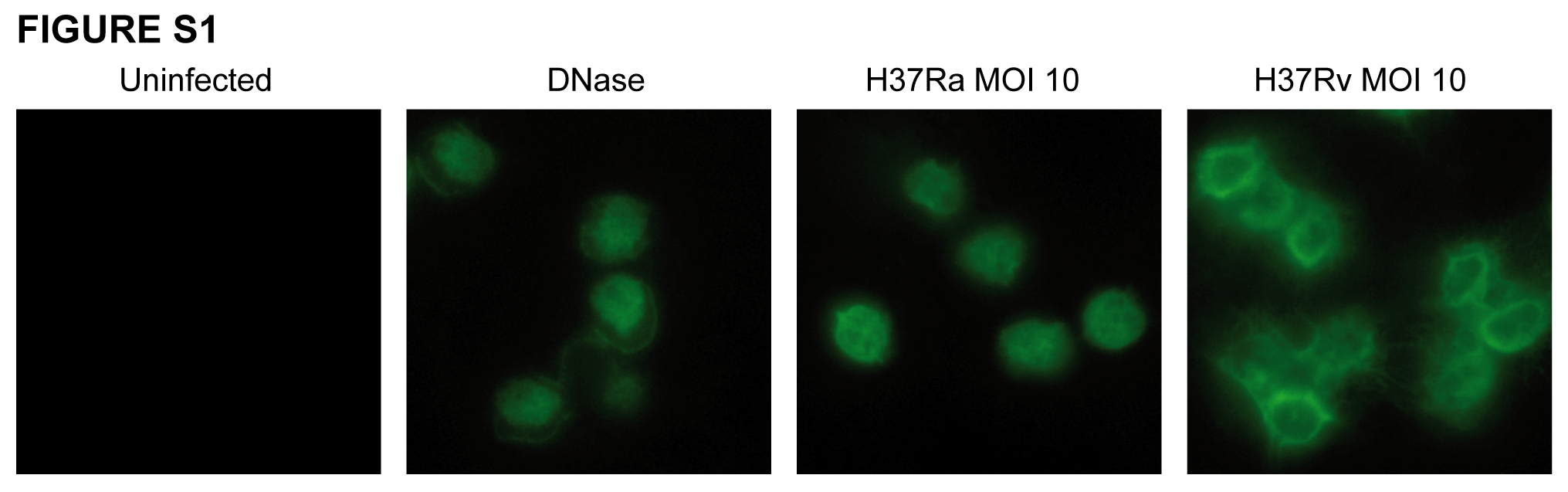

Supplement: Figure S1 — Fluorescence microscopy images of TUNEL-stained hMDMs. hMDMs on glass cover slips were infected with H37Ra or H37Rv at MOI 10, or left uninfected, fixed after two days of infection, and fragmented DNA was visualized by TUNEL staining. As a positive control, the hMDMs were treated with DNase prior to staining. Images were acquired using a fluorescence microscope, with the same settings for all samples. The images are representative of two independent experiments. (TIF) [file pone.0020302.s001.tif]

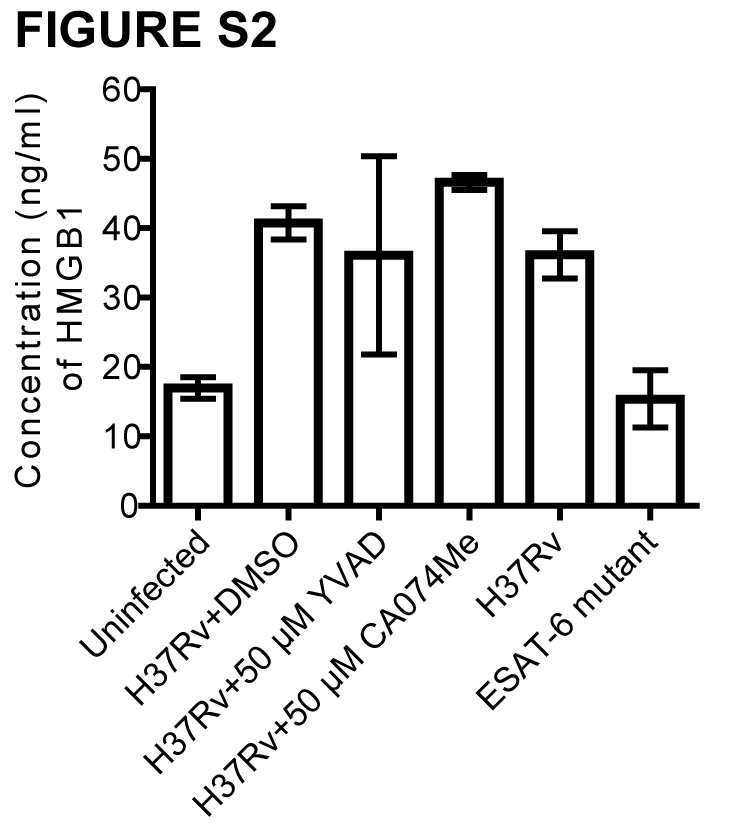

Supplement: Figure S2 — Increased HMGB1 release upon H37Rv infection is independent of caspase-1 and cathepsin B, but dependent on an intact ESAT-6 gene. hMDMs treated with DMSO (1:1000), YVAD, or CA-074Me were infected with H37Rv for two days at MOI 10. Alternatively, hMDMs were left uninfected or were infected at MOI 10 for two days with H37Rv or an ESAT-6 deletion mutant. The cell culture supernatants were then assayed for HMGB1 using ELISA (n = 3). The bar graph shows the mean HMGB1 concentration and the SEM. (TIF) [file pone.0020302.s002.tif]

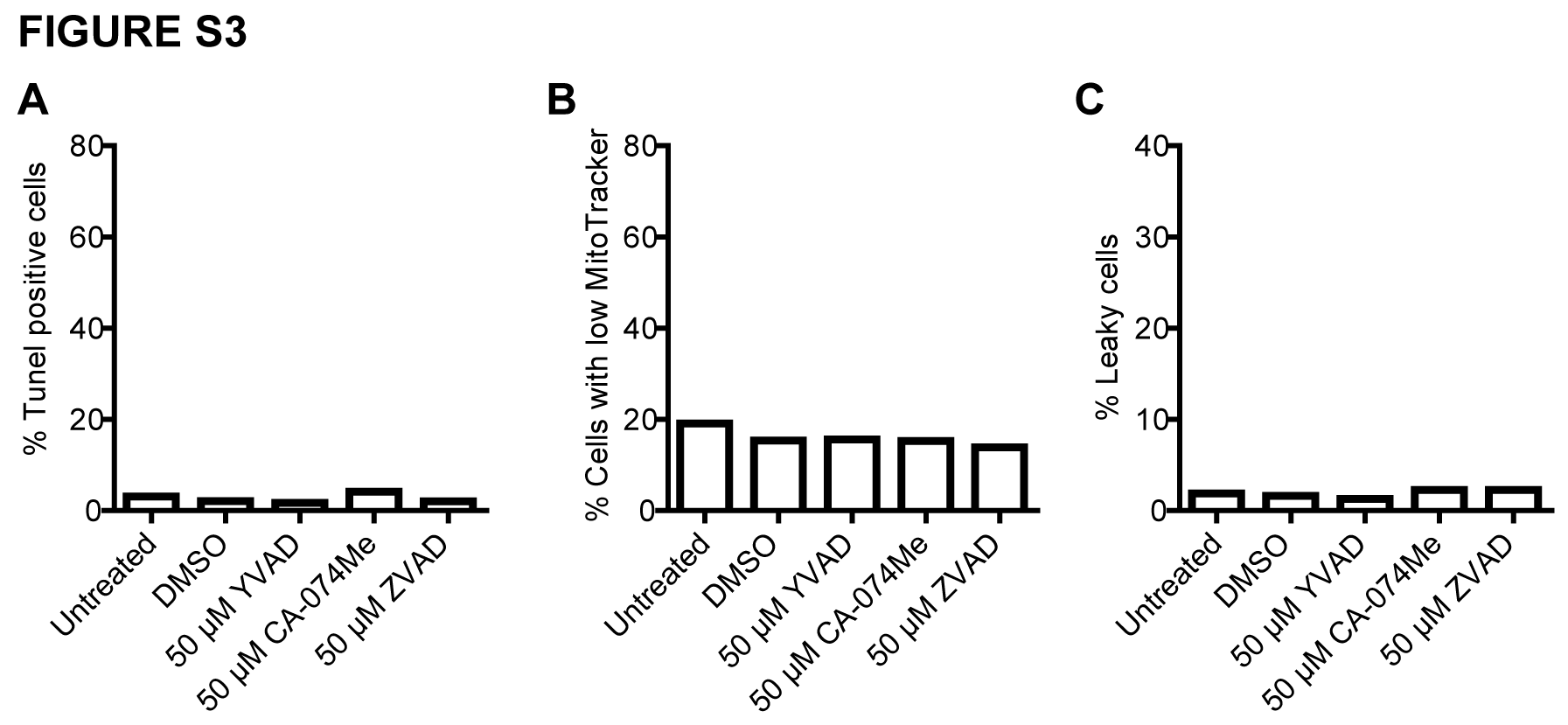

Supplement: Figure S3 — YVAD, CA-074Me and ZVAD do not affect macrophage viability. hMDMs were left untreated or were treated for 24 h with DMSO (1:1000) or the inhibitors at the concentration used in the manuscript. Three different staining procedures were used to investigate cell death features, and the cells were analyzed by flow cytometry. A) TUNEL analysis of DNA fragmentation. The bar graph shows the percentage of hMDMs that were positive for TUNEL staining (n = 1). B) MitoTracker analysis of mitochondrial membrane potential loss. The bar graph shows the percentage of hMDMs that had low MitoTracker staining (i.e. compromised mitochondria) (n = 1). C) Plasma membrane integrity analysis. The bar graph shows the percentage of hMDMs that were positive for plasma membrane leakiness staining (n = 1). (TIF) [file pone.0020302.s003.tif]

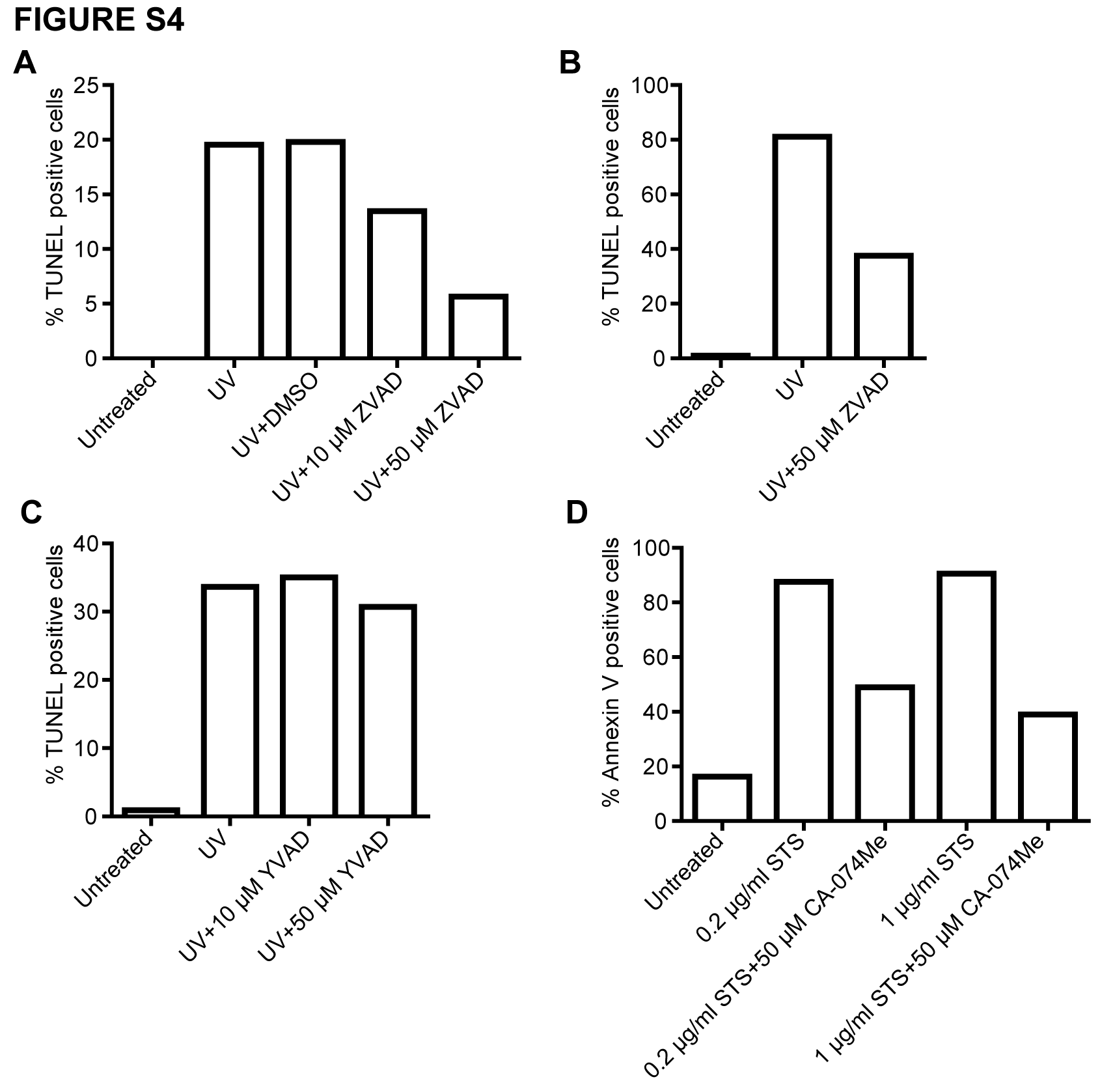

Supplement: Figure S4 — ZVAD and CA-074Me, but not YVAD, reduce UV- or staurosporine-induced apoptosis. hMDMs were treated with DMSO (1:1000) or inhibitors at the indicated concentration, and apoptosis was induced by UV light or by treatment with staurosporine for 18 h at the indicated concentration. Apoptosis was then assessed by TUNEL or Annexin V staining. A) Microscopy-based TUNEL analysis of DNA fragmentation after ZVAD and UV treatment. The bar graph shows the percentage of hMDMs that were positive for TUNEL staining (n = 1). B) Flow cytometry-based TUNEL analysis of DNA fragmentation after ZVAD and UV treatment. The bar graph shows the percentage of hMDMs that were positive for TUNEL staining (n = 1). C) Flow cytometry-based TUNEL analysis of DNA fragmentation after YVAD and UV treatment. The bar graph shows the percentage of hMDMs that were positive for TUNEL staining (n = 1). D) Flow cytometry-based Annexin V analysis of phosphatidyl serine externalization after CA-074Me and staurosporine (STS) treatment. Staining was performed using TACS Annexin V-FITC Apoptosis Detection Kit (R&D Systems) according to the manufacturer’s instruction. The bar graph shows the percentage of hMDMs that were positive for Annexin V staining (n = 1). (TIF) [file pone.0020302.s004.tif]

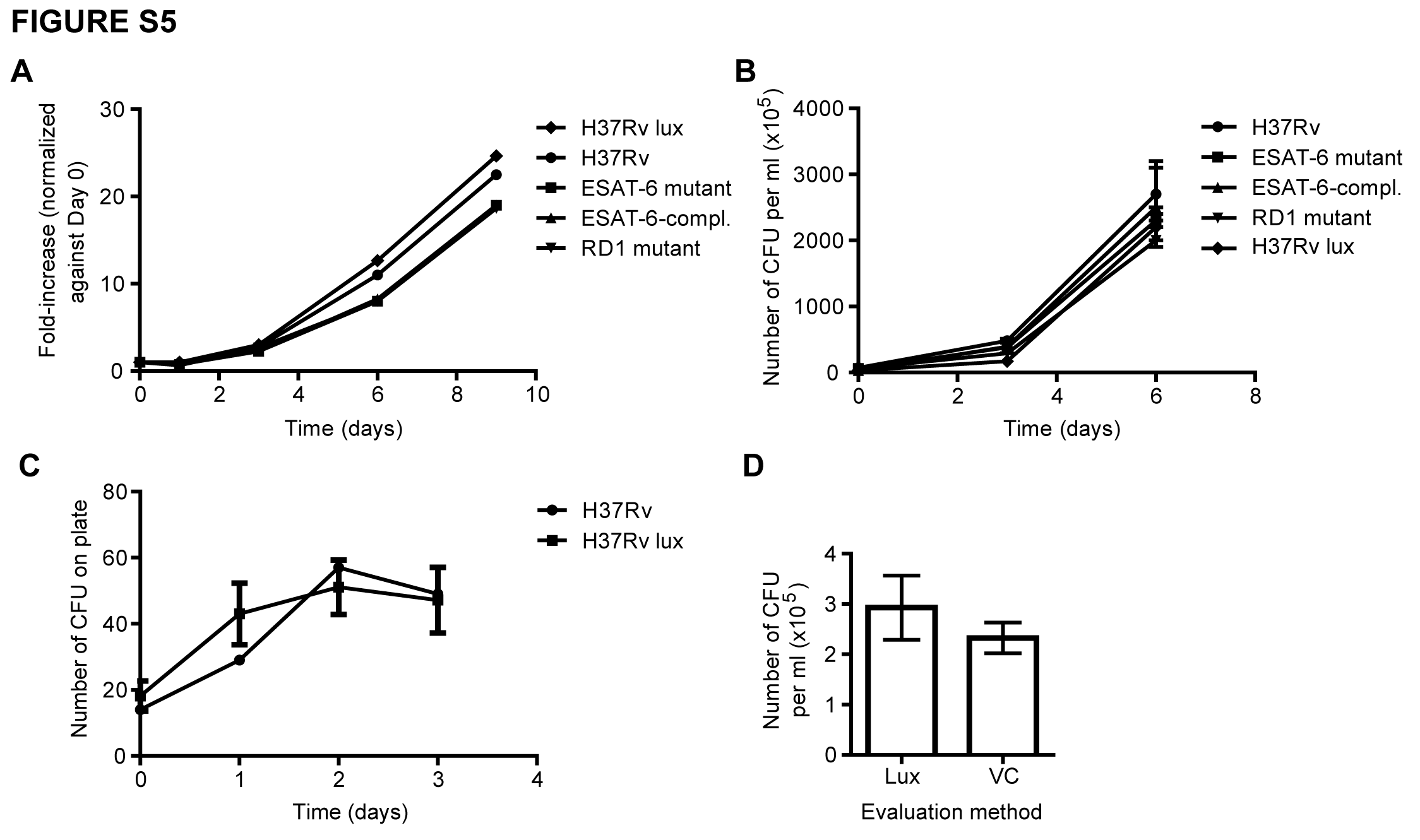

Supplement: Figure S5 — Similarity in growth between Mtb strains, and correlation between viable count and luciferase activity upon phagocytosis. A) 7H9 broth was inoculated with the different strains and the increase in bacterial numbers was assessed by OD600 at the indicated time points. The OD value was normalized against the Day 0 value for each time point and strain (n = 1). B) 7H9 broth was inoculated with the different strains, serial dilutions were plated at the indicated time points, and the number of CFU was enumerated after 2-3 weeks. The mean and SEM of triplicate plates is shown (n = 1). C) hMDMs were infected with H37Rv or luciferase-expressing H37Rv (H37Rv lux) at MOI 10, the macrophages were lysed, diluted and plated at the indicated time points, and the number of colonies on the plate was determined after 2-3 weeks. For H37Rv lux, the mean and SEM is shown (n = 6), and for H37Rv, one experiment is shown. D) hMDMs were infected with H37Rv lux at MOI 1, the macrophages were lysed after 4 h, and the number of CFU in the sample was determined by measuring luminescence (Lux) and converting the value to CFU, or by plating and determining the number of CFU by viable count (VC). The mean and SEM of the triplicates is shown (n = 1). (TIF) [file pone.0020302.s005.tif]
